# Are current seed storage approaches suitable for *Macrozamia fraseri* (Cycadales), a temperate species used in restoration?

**DOI:** 10.1093/conphys/coad096

**Published:** 2023-12-12

**Authors:** Shane R Turner, Simone Pedrini, Michael Just, Damian Grose, David Willyams, Kingsley W Dixon

**Affiliations:** ARC Centre for Mine Site Restoration, Curtin University, Kent Street, Bentley, Western Australia 6102, Australia; School of Molecular and Life Sciences, Curtin University, Kent Street, Bentley, Western Australia 6102, Australia; ARC Centre for Mine Site Restoration, Curtin University, Kent Street, Bentley, Western Australia 6102, Australia; School of Molecular and Life Sciences, Curtin University, Kent Street, Bentley, Western Australia 6102, Australia; ARC Centre for Mine Site Restoration, Curtin University, Kent Street, Bentley, Western Australia 6102, Australia; School of Molecular and Life Sciences, Curtin University, Kent Street, Bentley, Western Australia 6102, Australia; Tranen Revegetation Systems, Vincent Street, Bayswater Western Australia 6053, Australia; Plant Science & Environmental Technologies, Abeona Parade, Madora Bay, Western Australia, 6210, Australia; ARC Centre for Mine Site Restoration, Curtin University, Kent Street, Bentley, Western Australia 6102, Australia; School of Molecular and Life Sciences, Curtin University, Kent Street, Bentley, Western Australia 6102, Australia

**Keywords:** Conservation, drought stress, ex situ conservation, germination, non-orthodox, recalcitrant, seed ecology, seed storage, threatened flora

## Abstract

In this study, we focused on understanding key storage traits of seeds from *Macrozamia fraseri*, an unusual though important species that is impacted by mining. To support current restoration activities, large amounts of seed from *M. fraseri* have been regularly collected and stored for up to 8 years under standard seed banking conditions (5°C and 20% relative humidity), though *in situ* recruitment from directly sown seed is poor. To investigate the underlying constraints to germination on demand, we set out to assess the viability of *M. fraseri* seeds that had been stored in a restoration seed bank from 6 to 66 months. Seed moisture content (MC) (fresh weight basis) was also determined for seeds with different storage histories to ascertain whether *M. fraseri* seeds display traits (i.e. high MC) that might suggest non-orthodox seed storage behaviour. The youngest seed accession (6 months old) was found to have a high MC (45.8 ± 5.4%—fresh weight basis), and >50% viability. In comparison, older (>30 months old) accessions were observed to have a marked reduction in both seed MC (10–35% MC) and viability (0–29.4%). While preliminary, we conclude that *M. fraseri* seeds appear to lose viability during conventional storage with younger accessions displaying both a higher seed MC and viability, compared to accessions stored for longer. Given the significance of these results, future research activities are recommended to better understand the interplay between seed MC and storage environment and how this relates to the seasonally dry Mediterranean climate where this species naturally occurs. As well, storage and propagation approaches are proposed to increase success when using *M. fraseri* for conservation and restorative activities.

## Teaser

The implementation of effective storage techniques is a priority for seed banks. In this pilot investigation, we focused on understanding key traits of stored seeds from an endemic cycad impacted by mining. Preliminary results found that seeds in storage for longer displayed lower viability, suggesting that alternative seed banking approaches need consideration.

## Introduction

*Macrozamia* (Zamiaceae) is a common understory genus found in many regions of Australia with >40 species recognized (Atlas of Living Australia, 2023). Of concern is the fact that 10 of these are classified as either threatened or vulnerable (~25% of all known *Macrozamia* species), thus in need of conservation actions including *ex situ* storage (Martyn Yenson *et al.*, 2021; Department of Climate Change, Energy, the Environment and Water, 2023). Three closely related species of *Macrozamia* are found throughout southwestern Australia, a region with a strong seasonally dry Mediterranean climate (Groom and Lamont, 2015). Both *M. fraseri* and *Macrozamia riedlei* are key framework species within different native woodland communities throughout this region, which has experienced extensive clearing due to various activities such as urbanization, agriculture and mining (Beresford, 2001; Koch, 2007; Turner *et al.*, 2022).

For decades now, resource companies such Alcoa Australia and Hanson Australia have collected, stored and utilized tons of *Macrozamia* seed annually as part of post-mining rehabilitation works across this region, placing significant collection pressure on local *Macrozamia* populations already impacted by clearing and climate change (Beresford, 2001; Norman and Mullins, 2005; McFarlane *et al.*, 2020; Turner *et al.*, 2022). Multiple investigations have reported that *Macrozamia* seeds are slow to germinate and show a marked reduction in germination vigour if stored >12 months, although the reasons for this decline are poorly understood (Baird, 1939; Norman and Mullins, 2005; Turner *et al.*, 2022). Remarkably, *Macrozamia* seeds have also been demonstrated to be capable of germination when stored dry at room temperature, strongly suggesting seeds possess sufficient endogenous moisture to initiate and sustain the germination process in the absence of rainfall (Baird, 1939; Norman and Mullins, 2005; Turner *et al.*, 2022). Even so, *M. riedlei* seeds also reportedly show some capacity to emerge under field conditions (33%) after 2 years of prior *ex situ* storage, though higher emergence success (46%) was demonstrated for 1-year-old seed (Norman and Mullins, 2005). To the best of our knowledge, traits indicating non-orthodox seed storage behavior have never previously been either described or suggested for *M. fraseri* or the sympatric *M. riedlei*, and we are aware of at least four different resource companies collecting significant quantities of wild seeds from both species for use in post-mining restoration (Koch, 2007; Turner *et al.*, 2022).

*Macrozamia fraseri* is considered a priority species for the reestablishment of coastal plain woodlands and heathlands following mining as it is an important food source for vertebrates such as grey kangaroos (*Macropus fuliginosus*) and emus (*Dromaius novaehollandiae*) (Burbidge and Whelan, 1982; Groom and Lamont, 2015; Turner *et al.*, 2022). For example, Hanson Australia (Heidelberg Cement group) currently store >100 kg of *M. fraseri* seeds in their restoration seed bank, with some accessions now >8 years old (Turner *et al.*, 2022). Prior to storage, seeds are processed to remove the fleshy outer layer (sarcotesta) then stored at 5°C and 20% relative humidity (RH) until removed for use in direct seeding programs (Turner *et al.*, 2022). Nevertheless, germination results to date have been poor, with only a small percentage of *M. fraseri* seeds converting into plants. Baird, (1939) provided a detailed account of the reproductive biology of the closely related *M. reidlei*, which is likely to be similar to *M. fraseri* given that both species possess similar plant traits and overlap in parts of their ranges (Marchant *et al.*, 1987; Atlas of Living Australia, 2023). The embryo of *M. reidlei* is reported to be initially tiny when the seed is recently shed in late summer/early autumn (February/March) with germination (i.e. coleorhiza extension) not commencing until ~5 months later in autumn (April/May) the following year (Baird, 1939). As well, Baird, (1939) observed that the embryo slowly grows and matures within the seed for >12 months under field conditions, with much of this development occurring during the warmer drier summer months when rainfall is largely absent and the risk of desiccation assumed to be high (Groom and Lamont, 2015).

The current work aimed to inform and improve the management practices of a restoration seed bank when working with *M. fraseri* seeds through a detailed audit of seed accessions stored for different lengths of time. Consequently, we set out to determine whether seeds stored for up to 66 months under standard seed banking conditions were viable using a range of different techniques including X-ray assessment, floatation, cut test and tetrazolium test. Seed moisture content (MC) was determined for seeds with different storage histories and attributes to ascertain whether *M. fraseri* seeds display traits that might indicate non-orthodox seed storage behaviour. Based on these preliminary results, future research priorities are recommended as well as storage and propagation approaches that should be adopted to increase success when using *M. fraseri* (and related species) for both conservation and restoration activities.

## Materials and Methods

### Seed collection, processing and storage

**Figure 1:**
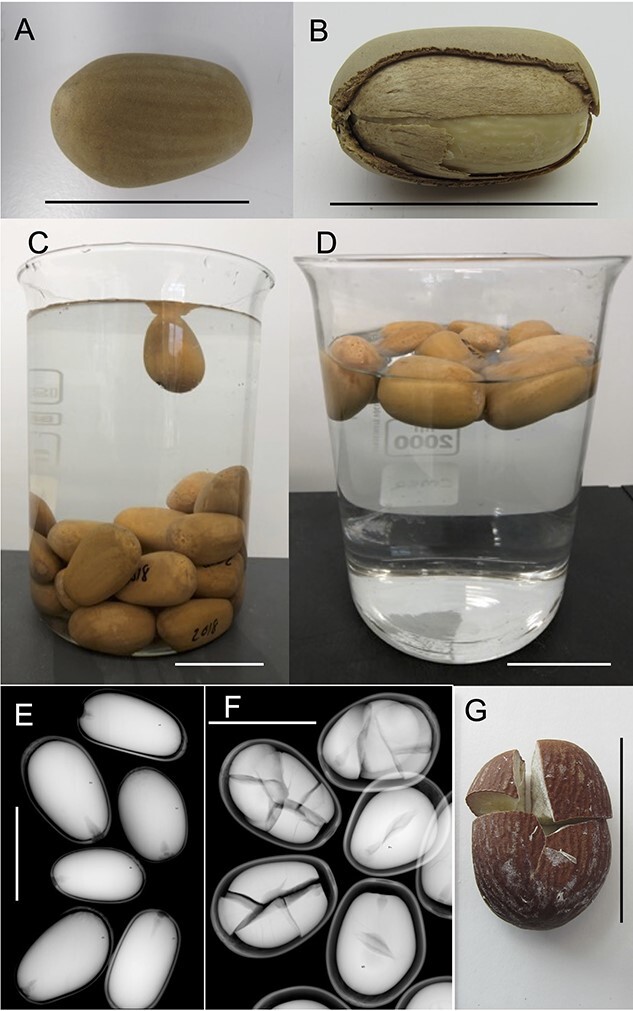
**A**) Intact sclerotesta of *M. fraseri* following manual removal of the outer pulpy sarcotesta using a rotating cement mixer with crushed rock aggregate and water; **B**) Longitudinally dissected sclerotesta showing the location and structure of the underlying thin membranous endotesta and the inner white megagametophyte; **C**) 30-month-old seeds stored under standard seed bank conditions (~20% RH and 5°C) subjected to a floatation test for 5 min; **D**) 54-month-old seeds stored under standard seed bank conditions subjected to a floatation test for 5 min; **E**) X-ray image of seeds stored for 6 months showing fully formed megagametophyte tissue within each seed with minimal signs of shrinkage or damage; **F**) X-ray image of seeds stored for 66 months showing fragmentation and significant megagametophyte shrinkage and **G**) Shattered megagametophyte following extraction from the sclerotesta as observed in Figure 1F. Line = 40 mm.

Seed was collected from shedding cones within 3 km of the Hanson Gaskell Sand Plant (31°45′06” S & 115°56′59″ E) between February 2015 and February 2020 by commercial seed contractors (Tranen Revegetation Systems—www.tranen.com.au) on behalf of Hanson Australia for use in *Banksia* woodland restoration following sand mining. *Macrozamia fraseri* seeds are large (~18 g per seed) and consist of a three-layered integument—a brightly coloured (red) outer fleshy layer (sarcotesta), an underlying hard layer (sclerotesta) and an inner thin membranous layer (endotesta) that surrounds the megagametophyte, the inner tissue that contains the main nutritive reserves in seeds of gymnosperms (Linkies *et al.*, 2010) (Figure 1A & B). To remove the outer pulpy layer (sarcotesta), a cement mixer, along with water and aggregate (10- to 30-mm crushed rock) was used. The mechanical action of the mixer, combined with the abrasiveness of the aggregate, separates the pulp from the sclerotesta (Figure 1A). The cement mixer was run for ~30 min and contained a slurry consisting of the freshly collected seeds, aggregate and water. Once the pulpy layer was removed, the slurry mix was placed into a large coarse sieve and washed thoroughly with clean water to wash through all the smaller debris, aggregate particles and pulp residue. After rinsing, the cleaned seeds were spread out in trays and placed in the sun for 7 days. The sclerotesta, endotesta and megagametophyte (hereafter collectively referred to as the ‘seed’—Figure 1B) were then placed into large porous woven polypropylene or calico bags and stored on large open trays in a temperature (~5°C) and RH (~20%) controlled environment room within the Tranen Restoration Seedbank (Turner *et al.*, 2022). Seeds were removed in September 2020 for use in this study.

Six different age classes (6, 18, 30, 42, 54 and 66 months) were assessed during this investigation, which were all considered single accessions per harvest year, with the oldest accession by this time having been in cold dry storage for ~66 months (collected in February 2015), while the youngest had been in storage for ~6 months (collected in February 2020). Approximately 500 g of seeds (26–34 seeds per accession) were provided by Tranen for research purposes from each of the six different age classes studied.

### X-ray assessment of seed fill

Determination of seed fill was performed by subjecting all seeds to X-ray imaging (VersaVision Digital Specimen Radiography System, Faxitron, AZ, USA). The resulting images were then analysed to determine the precise number of seeds that were either filled or structurally compromised (i.e. empty or internally cracked/shattered).

### Floatation and seed mass assessment

Previous research indicated that floatation can be an effective way to separate viable seeds (sinkers) from non-viable seeds (floaters) in some species of Cycad including *Cycas revoluta* and *Cycas taiwaniana* (Dehgan and Schutzman, 1989; Calonje *et al.*, 2011). To test this technique, seeds from each accession were placed into a 2000-ml beaker containing water for 5 min (Figure 1C & D) to determine the proportion that either floated or sank for each age class. Seeds were then removed from the water and individually weighed to quantify the average seed mass per age class, grouping together those seeds that sank and those that remained buoyant.

### Extraction and visual assessment

Megagametophytes (Figure 1B) were carefully removed from all the seeds used in the floatation test by gently fracturing the hard sclerotesta (Figure 1A) using a bench vice to apply even pressure along the seed’s longitudinal axis until it visibly cracked releasing the enclosed megagametophyte. Seeds that had shattered megagametophytes (Figure 1F & G) were recorded and excluded from further analysis. Megagametophytes that were intact (i.e. not cracked or shattered) were firstly imaged, then destructively assessed to determine their MC gravimetrically (see below).

### Seed moisture content

Initial megagametophyte mass (MM) and oven dry mass (ODM) were determined to calculate the average megagametophyte moisture content (MMC) for intact megagametophytes from each age class, grouping together those that sank and those that remained buoyant. To determine MMC, intact megagametophytes were initially weighed when removed from seeds, dried for 17 h at 103°C (International Seed Testing Association, 1999), then re-weighed within 10 min of removal from the drying oven with the difference in weight used to calculate MMC. Average MMC was determined for sinkers and floaters in each age class using 1–12 intact megagametophytes and is expressed on a fresh weight basis (International Seed Testing Association, 1999) to the nearest 0.1%.

### Embryo length and viability assessment

Average embryo length (in millimetres), megagametophyte length (in millimetres), embryo to megagametophyte (E:M) ratio and viability were determined on five seeds from each of four age classes (6–42 months) using only those seeds that had sunk (Figure 1C) and appearing to have fully formed white and unblemished megagametophyte tissue upon extraction and visual assessment (Figure 1B). Viability was estimated using both a cut test (Turner *et al.*, 2005) and 0.5% (w/v) 2,3,5-triphenyltetrazolium chloride (TZ) (Lakon, 1949). For the cut test, seeds were firstly dissected longitudinally, then visually inspected under a binocular microscope to ascertain whether there was any visible internal damage to either the embryo or surrounding megagametophyte tissue. Megagametophytes with signs of damage were scored as non-viable. Dissected megagametophytes were then placed cut-side down onto germination papers irrigated with 0.5% (w/v) TZ and incubated in darkness at 30°C for 24 h (Merritt *et al.*, 2021b).

## Statistical analysis

All statistical analyses were conducted using R software (version 4.0.3). To investigate the association between storage duration and the proportion of seeds that were shattered versus intact, or that floated versus sank, a binomial GLM with a logit link function was fitted to each of the two datasets. To determine whether the E:M ratio changed significantly with increasing storage duration, a Gaussian GLM with an identity link function was fitted to the data. The significance of each predictor variable was evaluated using the ‘Anova’ function in the ‘car’ package, with a significance level of α = 0.05. For comparisons between MMCs, a Wilcoxon rank-sum test was used to compare the MCs of seeds that floated (n = 47) to seeds that sank (n = 20).

## Results

### X-ray assessment

Based on X-ray assessment, seed quality varied significantly across the different age classes though all seeds were found to have megagametophyte tissue present (Table 1). For the three most recent age classes (6, 18 and 30 months old), <15% of seeds were observed to have shattered megagametophyte tissue and generally appeared to be structurally uniform with minimal megagametophyte shrinkage or other damage noted (Figure 1E). Older age classes (42, 54 and 66 months old) had a progressively higher proportion of megagametophyte tissue that were shattered (35–66%) with evidence of significant megagametophyte shrinkage clearly visible (Figure 1F). Storage duration had a significant negative effect on the number of intact seeds (χ^2^ = 41.38, *P ≤* 0.0001). Extraction and visual assessment of shattered megagametophyte tissue confirmed that these were significantly damaged and clearly no longer viable (Figure 1G) when compared with white and unblemished megagametophytes (Figure 1B).

**Table 1:** Proportion (%) of *M. fraseri* seeds stored for 6–66 months (+ the date of collection) in a restoration seed bank that were filled (Figure 1E) or showed internal shattering (Figure 1F) of the megagametophyte

**Storage duration & collection date**	**Filled & intact (%)**	**Filled & internally shattered (%)**
6 months (Feb. 2020)	86.2 (n = 25)	13.8 (n = 4)
18 months (Feb. 2019)	100.0 (n = 29)	0.0 (n = 0)
30 months (Feb. 2018)	100.0 (n = 26)	0.0 (n = 0)
42 months (Feb. 2017)	64.7 (n = 22)	35.3 (n = 12)
54 months (Feb. 2016)	62.5 (n = 20)	37.5 (n = 12)
66 months (Feb. 2015)	33.3 (n = 10)	66.7 (n = 20)

### Floatation and seed mass assessment

Seeds from the different age classes varied significantly in their capacity to either float or sink when immersed in water, with the older age classes (42–66 months old) having a significantly higher proportion (>70%) of seeds that remained buoyant compared with the younger age classes (<50%) (χ^2^ = 66.01, *P ≤* 0.0001) (Table 2). Interestingly, all the seeds that X-ray assessment had identified as possessing shattered megagametophyte tissues floated (Figure 1D). The average seed weight of all the seeds that were buoyant regardless of age class was >3 g less (15.4 ± 2.3 g, n = 107) than those that sank (18.8 ± 2.5 g, n = 67).

**Table 2:** Proportion (%) of *M. fraseri* seeds stored for 6–66 months in a restoration seed bank that either sank (Figure 1C) or floated (Figure 1D) when immersed in water for 5 min

	**Immersion response (%)**	
**Storage duration**	**Sinkers**	**Floaters**
6 months	58.6 (n = 17)	41.4 (n = 12)
18 months	72.4 (n = 21)	27.6 (n = 8)
30 months	96.2 (n = 25)	3.8 (n = 1)
42 months	29.4 (n = 10)	70.6 (n = 24)
54 months	0.0 (n = 0)	100.0 (n = 32)
66 months	0.0 (n = 0)	100.0 (n = 30)

### Extraction, visual assessment and seed moisture content

**Figure 2:**
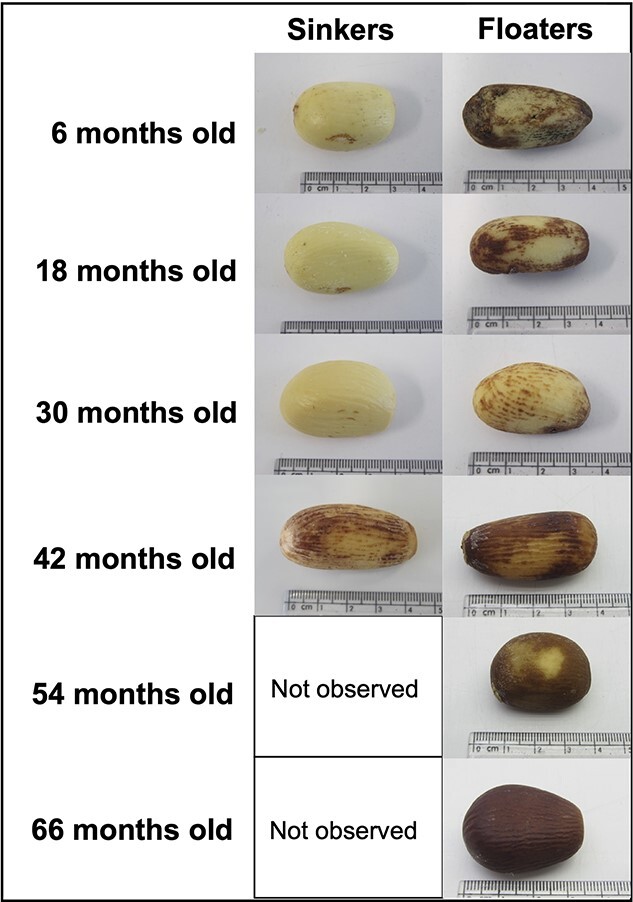
Extracted megagametophytes from *M. fraseri* seeds stored for 6–66 months at 5°C and 20% RH showing a range of attributes and damage such as discolouration and browning. For each age class, seeds were initially subjected to a floatation test to separate sinkers from floaters, then megasporophytes manually extracted, visually assessed, weighed and placed into an oven to determine MMC. It was noted that most seeds that sank were white and intact (healthy) with minor browning appearing in the 42-month age class, while those that floated were all found to have various levels of damage with none determined to be viable, i.e. uniformly white and unblemished.

Megagametophytes extracted from buoyant seeds were observed to show various signs of tissue damage and degradation such as blackening and browning, with the older age classes (42–66 months) showing considerably more damage than the younger age classes (Figure 2). In comparison, megagametophytes derived from seeds that had sunk appeared white and healthy and were generally free from discolouration and obvious signs of tissue damage and degradation (Figure 2).

**Figure 3:**
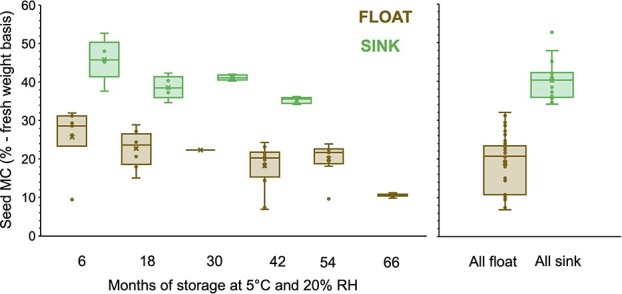
Box and whisker plots of *M. fraseri* MMC showing the minimum, maximum, median, mean (x) and 25–75% quartile range calculated on a fresh weight basis. Data were derived from assessment of intact seeds that either floated or sank for each of the six storage periods (left graph) or when all storage durations were pooled into two classes, i.e. those that floated or those that sank (right graph).

MMC varied considerably, with the highest MC (45.8 ± 5.4%) derived from seeds from the youngest age class (6 months) that had sunk (Figure 3), with all of these appearing to be white and healthy (Figure 2—top row left). The lowest MMC (10–20%) recorded, were derived from megasporophytes from the three oldest age classes (42–66 months) that had floated (Figure 3), all of which showed significant signs of tissue degradation and browning (Figure 2—bottom three rows right). When pooled, the average MMC of all (n = 20) the sinkers was >2-fold higher (40.2 ± 1.1%) than all (n = 47) the floaters (18.9 ± 1.0%) (Figure 3) (*P* ≤ 0.0001).

### Embryo length and viability assessment

Megagametophytes derived from seeds that had sunk were observed to be 39–47 mm in length and possess underdeveloped embryos 5–7 mm long (Table 3). The E:M ratio was calculated to be 0.12–0.16 across all four storage durations assessed (χ^2^ = 3.3942, *P* = 0.06; Table 3).

**Table 3:** Average megagametophyte length (in millimetres), embryo length (in millimetres), E:M ratio and viability (%) based on a cut test and 0.5% (w/v) TZ assessment of *M. fraseri* seeds stored for 6–42 months in a restoration seed bank

**Storage duration (approx.)**	**Megagametophyte length (mm ± SE)**	**Embryo length (mm ± SE)**	**E:M ratio (± SE)**	**Viability (%) (TZ/cut test)**
~6-months-old	40.8 ± 3.1 (n = 2)	6.6 ± 2.3 (n = 2)	0.16 ± 0.05 (n = 2)	40 (n = 2)
~18-months-old	39.6 ± 2.5 (n = 5)	7.0 ± 1.6 (n = 5)	0.18 ± 0.03 (n = 5)	100 (n = 5)
~30-months-old	43.6 ± 2.1 (n = 5)	6.8 ± 0.8 (n = 5)	0.16 ± 0.01 (n = 5)	100 (n = 5)
~42-months-old	47.4 ± 1.9 (n = 5)	5.8 ± 1.6 (n = 5)	0.12 ± 0.03 (n = 5)	100 (n = 5)

**Figure 4:**
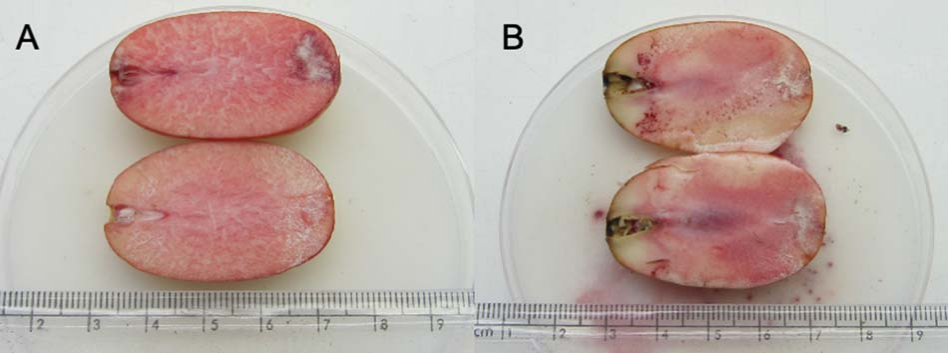
A) Longitudinally dissected megagametophyte of *M. fraseri* stored for 6 months showing the results of TZ assessment—both the cut test and TZ assessment indicated that this megagametophyte + embryo were viable; B) Longitudinally dissected megagametophyte stored for 6 months showing the results of TZ assessment—both the cut test and TZ assessment indicate that this megagametophyte + embryo were not viable due to the internal browning within the embryo region and lack of TZ staining in this area.

Cut test and TZ assessment found that megagametophytes from the 18-, 30- and 42-month storage classes that had sunk were deemed to be 100% viable with no signs of internal damage when dissected, with both the embryo and megagametophyte uniformly staining red in the presence of TZ (Figure 4A). However, viability for the 6-month storage class was calculated to be only ~40% as seed dissection and careful observation of embryos found that two had internally rotted (i.e. were brown/grey in appearance rather than white), while the surrounding megagametophyte tissue still appeared to be healthy, which was also confirmed with the lack of TZ staining immediately around the embryo region (Table 3; Figure 4B).

## Discussion

The seeds of zamia (*M. fraseri* and *M. riedlei*) are clearly unusual when compared with the seeds of other sympatric species found in the Mediterranean-type climate region of the southwest Western Australian biodiversity hotspot (Merritt *et al.*, 2007; Dalziell *et al.*, 2022). For example, of the 8600 species in this floristic region, zamia possess the largest seeds by far for any species (TSW (thousand seed weight) = 18 000 g), and globally are one of the largest seeds recorded, falling into the top 1% of species in terms of seed mass (Moles *et al.*, 2005). Further, germination is exceptionally slow, taking >12 months to occur, which is attributable to an underdeveloped embryo (E:M ratio = ~0.15—Table 3) that requires considerable time to grow and mature within the seed prior to coleorhiza emergence (Baird, 1939; Norman and Mullins, 2005). Finally, germination has been demonstrated to occur in the absence of water with air-dried laboratory-stored seeds commencing germination many months after collection (Baird, 1939; Norman and Mullins, 2005). Taken together, these unusual seed attributes have considerable management implications for the use of *M. fraseri* seeds in restoration as the usual seed-handling approaches recommended for orthodox-seeded species are clearly inadequate and require modification to improve the likelihood of success when working with this species (Norman and Mullins, 2005; Turner *et al.*, 2022).

Current recommendations for storing orthodox seeds to maintain a high level of viability for up to 10 years involve placement in a cool (5–20°C), dry (10–25% RH) environment to facilitate initial seed drying, which consequently impedes seed deterioration (FAO, 2014; Merritt *et al.*, 2021*a*). In low-RH environments, freshly mature desiccation-tolerant seeds with a relatively high MC (<20%) gradually lose moisture, eventually reaching equilibrium ~3–7% seed MC usually within 1–4 weeks (De Vitis *et al.*, 2020). By comparison, many of the *M. fraseri* seeds assessed in this study, (even after 42 months of storage), were found to possess considerably higher moisture levels (>35%), suggesting that their physiology is noticeably different from most other species, although the specific reasons for this distinction remain unclear.

The floatation test proved to be a surprisingly simple and effective way to separate viable *M. fraseri* seeds (sinkers) from non-viable seeds (floaters) (Table 2). Floatation has previously been found to be an effective way to separate viable from non-viable seeds across a broad range of species, including species of cycad (Dehgan, 1983). However, for *Cycas rumphii*, *Cycas seemanii*, *Encephalartos arenarius* and *Encephalartos nubimontanus* (Calonje *et al.*, 2011) the floatation test has proven ineffective, indicating that calibration with another method (such as X-ray or a cut test) is required when using seed floatation for the first time. Regardless of storage duration (6–66 months), every *M. fraseri* seed that floated here in this study was observed to be clearly non-viable when assessed (Figure 2). In comparison, all seeds that sank appeared healthy, possessing both fully formed white megagametophyte tissue and a high (>35%) MC (Figure 2). Indeed, seed MC among different age classes was much higher than expected, with 6-month-old seeds possessing an MC of 45.8 ± 5.4% (fresh weight basis) (Table 2). It must be noted that by the time of assessment, these seeds had been in cool dry storage for ~6 months, and it is possible that the MC was higher at seed dispersal. Seed MC was also found to be lower (10–20%—fresh weight basis) for accessions both older than 42 months and observed to float when placed in water. It was also determined that all seeds in this group were also non-viable (Table 1; Figs. 1 & 2). Unsurprisingly, buoyant seeds were also lighter overall (15.4 ± 2.3 g) than those that sank (18.8 ± 2.5 g), which we attribute to the loss of moisture during storage and the resultant megagametophyte shrinkage observed in the X-ray assessment of buoyant seeds (Figure 1F).

There was a strong relationship between storage duration and the proportion of seeds that showed evidence of either megagametophyte shrinkage or internal shattering (Table 2), with seeds stored for ≥42 months showing significant signs of deterioration, and in most cases, no longer appeared to be viable (Tables 1 & 2; Figure 2). Nevertheless, there was still substantial variability in seed quality between the three most recent age classes where most viable seeds were observed (i.e. 6-, 18- and 30-month storage durations) with the 6-month-old seeds showing reduced seed quality when compared with the two next oldest age classes (Tables 1 to 3). X-ray assessment failed to identify some of these non-viable seeds as their poor quality was only determined during TZ testing of internal megagametophyte tissues after dissection (Table 3; Figure 4B). It must be acknowledged that the TZ test can show a false-positive result on occasion for a range of reasons so without calibration (i.e. correlation with germination data), this result should be viewed with some level of caution (Merritt *et al*., 2021b). The embryo region in these seeds appear to have degraded for unknown reasons but may have happened either during seed development and maturation or possibly as a result of the initial cleaning treatment to remove the pulpy sarcotesta.

Of relevance to this study, it has been noted for both *M. riedlei* (Baird, 1939; Norman and Mullins, 2005) and *M. fraseri* (Turner unpublished results) that germination (i.e. coleorhiza extension) commences in the absence of moisture. This attribute is strikingly different from most seeds which require exposure to water to initiate the germination process and indicate that the seed biology of *M. fraseri* is fundamentally different from most other seed-bearing plants (Berjak and Pammenter, 2008). The moisture content (45.8 ± 5.4%) values of viable 6-month-old *M. fraseri* seeds neatly fall within the interquartile range (43–58%) of a broad range of desiccation-sensitive rainforest species (Sommerville *et al.*, 2021—Fig. 7A). Given the high level of congruence, these data suggest that seeds of *M. fraseri* may be non-orthodox, which if confirmed would be a highly unusual trait for seeds to possess in a Mediterranean climate (Wyse and Dickie, 2017). Mediterranean ecosystems are regularly subjected to warm to hot summer temperatures, drought and extended periods of low humidity; conditions not typically aligned with the presence of non-orthodox seeded species and the formidable challenges of surviving these conditions (Rundel *et al.*, 2016).

While more work is needed to thoroughly understand the underlying seed physiology of *M. fraseri*, we are confident based on the results in this pilot study that 1) mature seeds of zamia are likely shed with a high (>40%) moisture content and 2) preliminary evidence suggests that zamia seeds quickly lose viability while maintained under standard seed storage conditions (~5°C and <20% RH), which may be linked to a reduction in MC while in storage. Nevertheless, additional information is needed to comprehend how these seeds persist and function in a Mediterranean environment that is regularly subjected to months of warm to hot, dry conditions. It is clear from these results, that new approaches for *M. fraseri* are needed to improve seed storage outcomes and later propagation success. Consequently, while additional research is completed into establishing critical seed storage thresholds, we recommend that **1)** Floatation be used to separate viable (sinkers) seeds from non-viable (floaters) seeds; **2)***Macrozamia fraseri* seeds be used for the production of greenstock for later field planting rather than stored for future use; **3)** Seeds should be surface-sown under nursery conditions as soon as possible and exposed to natural temperatures and rainfall patterns rather than intense irrigation as seeds may rot, particularly when exposed to summer temperatures and; **4)** Avoid storing *M. fraseri* seeds under standard seed banking conditions (low temperature and RH) due to the possibility of desiccation damage that will inevitability compromise seed viability and as a consequence germination capacity.

## Data Availability

The data underlying this article will be shared on reasonable request to the corresponding author.

## References

[ref1] Atlas of Living Australia (2023) *Atlas of Living Australia*. http://www.ala.org.au (March 15, 2023 date accessed)

[ref2] Baird AM (1939) A contribution to the life history of *Macrozamia reidlei*. J R Soc West Aust 25: 153–175.

[ref3] Beresford Q (2001) Developmentalism and its environmental legacy: the Western Australia Wheatbelt, 1900–1990s. Aust J Politics Hist 47: 403–415. 10.1111/1467-8497.00236. 19112676

[ref4] Berjak P , Pammenter NW (2008) From *Avicennia* to *Zizania*: seed recalcitrance in perspective. Ann Bot 101: 213–228. 10.1093/aob/mcm168. 17704237 PMC2711015

[ref5] Burbidge A , Whelan R (1982) Seed dispersal in a cycad, *Macrozamia riedlei*. Aust J Ecol 7: 63–67. 10.1111/j.1442-9993.1982.tb01300.x.

[ref6] Calonje M , Kay J, Griffith M (2011) Propagation of cycad collections from seed: applied reproductive biology for conservation. Sibbaldia 9: 77–96.

[ref7] Dalziell E , Lewandrowski W, Commander L, Elliott C, Erickson T, Tudor E, Turner S, Merritt D (2022) Seed traits inform the germination niche for biodiverse ecological restoration. Seed Sci Technol 50: 103–124. 10.15258/sst.2022.50.1.s.06.

[ref8] De Vitis M , Hay FR, Dickie JB, Trivedi C, Choi J, Fiegener R (2020) Seed storage: maintaining seed viability and vigor for restoration use. Restor Ecol 28: S249–S255. 10.1111/rec.13174.

[ref9] Dehgan B (1983) Propagation and growth of cycads - a conservation strategy. Proceedings of the Florida State Horticultural Society 96: 137–139.

[ref10] Dehgan B , Schutzman B (1989) Embryo development and germination of cycas seeds. J Am Soc Hort Sci 114: 125–129. 10.21273/JASHS.114.1.125.

[ref11] Department of Climate Change, Energy, the Environment and Water (2023) EPBC ACT List of Threatened Species. *EPBC ACT List of Threatened Species*. http://www.environment.gov.au/cgi-bin/sprat/public/publicthreatenedlist.pl?wanted=flora (March 15, 2023 date accessed).

[ref12] FAO (2014) Genebank Standards for Plant Genetic Resources for Food and Agriculture. Rome, Food and Agriculture Organization of the United Nations

[ref13] Groom P , Lamont B (2015) Plant Life of Southwestern Australia Adaptations for Survival. Walter de Gruyter GmbH & Co KG, Warsaw/Berlin

[ref14] International Seed Testing Association (1999) International rules for seed testing. Seed Sci Technol 27: 1–333.

[ref15] Koch JM (2007) Restoring a jarrah forest understorey vegetation after bauxite mining in Western Australia. Restor Ecol 15: S26–S39. 10.1111/j.1526-100X.2007.00290.x.

[ref16] Lakon G (1949) The topographical tetrazolium method for determining the germinating capacity of seeds. Plant Physiol 24: 389–394. 10.1104/pp.24.3.389. 16654232 PMC437388

[ref17] Linkies A , Graeber K, Knight C, Leubner-Metzger G (2010) The evolution of seeds. New Phytol 186: 817–831. 10.1111/j.1469-8137.2010.03249.x. 20406407

[ref18] Marchant NG , Wheeler JR, Rye BL, Bennett EM, Lander NS, McFarlane TD (1987) Flora of the Perth Region, Part One. Herbarium, Department of Agriculture., Perth, Western Australia, Western Australian

[ref19] Martyn Yenson AJ , Offord CA, Meagher PF, Auld T, Bush D, Coates DJ, Commander LE, Guja LK, Norton SL, Makinson RO et al. (2021) Plant Germplasm Conservation in Australia: Strategies and Guidelines for Developing, Managing and Utilising Ex Situ Collections, Ed3rd. Australian Network for Plant Conservation, Canberra, Australia

[ref20] McFarlane D , George R, Ruprecht G, Charles S, Hodgson G (2020) Runoff and groundwater responses to climate change in South West Australia. J R Soc West Aust 103: 9–27.

[ref21] Merritt D , Cochrane A, Commander L, Sommerville K, Breman E, Quarmby A (2021a) Florabank Guidelines Module 9 – Seed drying and storage. In: Commander LE, ed. FloraBank Guidelines, Ed2nd. Australia, FloraBank Consortium.

[ref22] Merritt DJ , Turner SR, Clarke S, Dixon KW (2007) Seed dormancy and germination stimulation syndromes for Australian temperate species. Aust J Bot 55: 336–334. 10.1071/BT06106.

[ref23] Merritt DJ , Whitehouse KJ, Hoyle GL, Crawford A, Wood JA, Satyanti A, Norton SL, Errington G, Martyn Yenson AJ (2021b) Chapter 5 Seed banking: orthodox seeds. In: Martyn Yenson AJ, Offord CA, Meagher PF, Auld TD, Bush D, Coates DJ, Commander LE, Guja LK, Norton SL, Makinson RO, Stanley R, Walsh N, Wrigley D, Broadhurst L, eds. Plant Germplasm Conservation in Australia: Strategies and Guidelines for Developing, Managing and Utilising Ex Situ Collections, Ed3rd. Australian Network for Plant Conservation, Canberra, Australia, pp 119–157.

[ref24] Moles AT , Ackerly DD, Webb CO, Tweddle JC, Dickie JB, Westoby M (2005) A brief history of seed size. Science 307: 576–580. 10.1126/science.1104863. 15681384

[ref25] Norman MA , Mullins G (2005) Effect of Seed Age, Sowing Season and Burial Depth on Establishment of Zamia (Macrozamia riedlei) (No. 22). ALCOA Australia, Booragoon, WA, Australia.

[ref26] Rundel PW , Arroyo MTK, Cowling RM, Keeley JE, Lamont BB, Vargas P (2016) Mediterranean biomes: evolution of their vegetation, floras, and climate. Annu Rev Ecol Evol Syst 47: 383–407. 10.1146/annurev-ecolsys-121415-032330.

[ref27] Sommerville KD , Errington G, Newby Z-J, Liyanage GS, Offord CA (2021) Assessing the storage potential of Australian rainforest seeds: a decision-making key to aid rapid conservation. Biodivers Conserv 30: 3185–3218. 10.1007/s10531-021-02244-1.

[ref28] Turner SR , Cross AT, Just M, Newton V, Pedrini S, Tomlinson S, Dixon K (2022) Restoration seedbanks for mined land restoration. Restor Ecol 30: e13667. 10.1111/rec.13667.

[ref29] Turner SR , Merritt DJ, Baskin CC, Dixon KW, Baskin JM (2005) Physical dormancy in seeds of six genera of Australian Rhamnaceae. Seed Sci Res 15: 51–58. 10.1079/SSR2004197.

[ref30] Wyse SV , Dickie JB (2017) Predicting the global incidence of seed desiccation sensitivity. J Ecol 105: 1082–1093. 10.1111/1365-2745.12725.

